# Autologous CD33-CAR-T cells for treatment of relapsed/refractory acute myelogenous leukemia

**DOI:** 10.1038/s41375-021-01232-2

**Published:** 2021-04-08

**Authors:** Francesco Paolo Tambaro, Harjeet Singh, Emily Jones, Michael Rytting, Kris M. Mahadeo, Philip Thompson, Naval Daver, Courtney DiNardo, Tapan Kadia, Guillermo Garcia-Manero, Tim Chan, Rutul R. Shah, William G. Wierda

**Affiliations:** 1 Department of Pediatrics, The University of Texas MD Anderson Cancer Center, Houston, TX USA; 2 Unità Operativa di Trapianto di Midollo Osseo e Servizio Trasfusionale; Azienda Ospedaliera di Rilievo Nazionale Santobono-Pausilipon, Napoli, Italy; 3 Department of Leukemia, The University of Texas MD Anderson Cancer Center, Houston, TX USA; 4 Precigen, Inc.20358 Seneca Meadows Parkway, Germantown, MD USA

**Keywords:** Acute myeloid leukaemia, Diseases

## To the Editor:

Acute myelogenous leukemia (AML) is a potentially curable disease; 70% of newly diagnosed patients achieve complete remission with first-line therapy, but prognosis worsens for relapsed disease in both pediatric and adult patients. A recent study examined long-term overall survival (OS) in relapsed patients with AML enrolled to nine successive ECOG-ACRIN trials for newly diagnosed AML between 1984 and 2008. Importantly, a median OS of 6 months from relapse and 10% 5-year OS were reported [1]. Recently, encouraging results were reported for targeted agents against molecular markers associated to the poor prognosis in relapsed disease given as monotherapy or combined with chemotherapy, including FLT3- [2], IDH1- [3] and IDH2-inhibitors [4].

Remarkable results were achieved with CAR-modified T cells targeting CD19 in relapsed/refractory patients with diffuse large B cell lymphoma (DLBCL) [5] and pediatric B cell acute lymphoblastic leukemia (ALL) [6] (50–90% CR), which inspired development of CAR-T cellular therapy for other indications, such as AML. Identifying a myeloid target for CAR-T cellular therapy has proven challenging, since surface antigens are shared by malignant myelogenous cells and normal hematopoietic stem cells, potentially resulting in prolonged myelotoxicity when tested in clinical trials. CD33, a member of the sialic acid-binding Ig-like lectin family, is expressed on subpopulations of leukemia cells, including leukemic stem cells, in almost 90% of AML cases as well as on some normal myeloid precursors [7].

We initiated a single-center, single-arm, Phase I clinical trial (NCT03126864) to investigate the feasibility and safety of autologous T cells, modified to express a CD33-targeted CAR with 4-1BB and CD3ζ endo-domains and co-expressed with truncated human epidermal growth factor receptor (HER1t) [8], in patients with relapsed/refractory AML. The goals of this Phase I clinical trial were to assess the feasibility and safety of adoptive transfer of the autologous CD33-CAR-T cells identify the recommended Phase II dose. Details of the clinical trial, including product release criteria (Table S1), can be found in the Supplementary Material.

Ten adults with relapsed/refractory AML were enrolled; 7 were male; median age was 30 (range 18–73) years (Table 1). Patients had received a median of 5 (range 3–8) prior treatment regimens; 3 underwent prior allogeneic stem cell transplant. The median percent bone marrow blasts at screening, WBC, the percent peripheral blood blasts and the lymphocyte count at apheresis were 55% (10–88), 1.6/µl (0.5–7.6), 26% (6–80), and 0.63/µl (0.01–1.5), respectively. Apheresis was collected for 8 patients; 4 had CD33-CAR-T cells produced which met release pre-specified release criteria for infusion; 4 had CD33-CAR-T cell product that failed to meet at least one pre-specified release criteria; and 2 patients had rapidly progressive AML and could not undergo apheresis (Table 2). Three patients received CD33-CAR-T cells at the first dose level (0.3 × 10^6^ CD33-CAR-T/kg) and 1 patient died before receiving their cells. The time from apheresis to CD33-CAR-T cell infusion is shown (Table 1).

**Table 1:** Patient characteristics and treatment.

Patient number	01	02	03	04	06	(05)07*	08	09	10	11
Sex	M	M	M	M	M	M	M	F	F	F
Age	36	73	38	18	18	18	52	24	56	19
Number Prior therapies	6	7	5	5	5	7	8	5	3	5
% BM Blasts at screening	10	18	50	60	47	86	27	66	60	85
Number WBC/μl at apheresis	0.8	5.4	1.7	0.9	0.5	7.6	5.5	na	na	1.5
Number lymphocytes/mL at apheresis (%)	0.16 (20)	1.5 (28)	0.48 (28)	0.6 (67)	0.01 (2)	0.76 (10)	0.66 (12)	na	na	0.92 (61)
% PB Blasts at apheresis	6	21	63	20	80	74	8	na	na	31
Apheresis	y	y	y	y	y	y	y	n	n	y
% T cells in apheresis product	14.6	33.5	23.2	45.7	46.4	4	7.1	na	na	64.6
% CD33^+^ blasts in apheresis product	54.7	24.4	60.9	45.4	34.2	94.6	ND	na	na	22.5
CD33-CAR-T cells product released (y/n)	n	y	y	n^#^	y	n	n	na	na	y
CD33-CAR-T cell infusion (y/n)	n	n	y	n	y	n	n	na	na	y
Apheresis to CD33-CAR-T infusion (days)	na	na	94	na	36	na	na	na	na	49
Dose (×10^6^/kg)	na	na	0.3	na	0.3	na	na	na	na	0.3

y, yes; n, no; na, not applicable; ND, not determined; #, number.

^*^ Pts 5 and 7 were same patient.

^#^ For Pt 4, sufficient CD33-CAR-T cells were manufactured but %CD3 + -CAR + was below release specification and product lot was successfully released per agreement with the FDA.

**Table 2:** Patient outcomes*.

Patient number	03						06						11					
Day of protocol	**Pre-LD**	**0**	**3**	**7**	**14**	**30**	**Pre-LD**	**0**	**3**	**7**	**14**	**30**	**Pre-LD**	**0**	**3**	**7**	**14**	**30**
WBC (/μl)	9.4	1.4	0.3	0.6	0.4	NT	0.6	0.6	0.5	0.4	0.2	4.2	1.0	0	0	0	0.1	0.4
% lymphocytes	22	28	NR	54	65	NT	57	40	51	65	NR	15	79	NR	NR	NR	NR	42
% CD33-CAR-T (blood flow HER1t)	NA	0.05	0	0.37	1.8	NT	NA	0.07	0.02	0.03	1.68	NT	NA	0	0.03	0.09	0.59	NT
CD33-CAR-T by ddPCR (HER1t copies/μg blood DNA)	NA	0	0	65	0	NT	NA	0	0	8961	3032	NT	NA	0	0	584	3701	NT
% Blood blasts	78	64	NR	44	35	NT	35	21	29	20	NR	78	12	NR	NR	NR	NR	38
CRS (Grade)	3 (D7)						0						2 (D6)					
ICANS (Grade)	2 (D9)						0						0					
AE – CD33-CAR-T-related (Grade)	TLS – AKI (G3) Mucositis (G2) Tachycardia (G1)						Intermittent Orthostatic Hypotension (G2) ↑ Bilirubin (G2) ↑ ALT (G3) ↑ AST (G3)						None					
AE – CD33-CAR-T-unrelated (Grade)	Respiratory Distress (G4)						None						Febrile Neutropenia (G3) Sepsis (G4) Septic Shock (G4)					
Cytokines: Baseline — Peak (Day)																		
IL6 (0–5 pg/mL)	69 **—** 28025 (D7)						0 **—** No change						10 **—** 330 (D10)					
IFNγ (0–5 pg/mL)	2 **—** No change						0 **—** No change						32 **—** 166 (D6)					
TNFα (0–22 pg/mL)	49 **—** 142 (D7)						0 **—** No change						40 **—** 886 (D6)					
Tocilizumab/Steroids	Y/Y						None						Y/N					
Day from CAR-T taken off study	D20						D29						D31					
Reason taken off study	Disease Progression						Disease Progression						Disease Progression					
Status	Deceased						Deceased						Deceased					

*^#^ number, *AE* adverse event, *AKI* acute kidney injury, *CD33-CAR-T* CD33-directed chimeric antigen receptor-T cell, *CRS* cytokine release syndrome, *D* day, *G* grade, *ICANS* immune effector cell-associated neurotoxicity syndrome, *N* no, *NA* not applicable, *NR* not reported, *NT* not tested, *Pre-LD* pre-lymphodepletion, *TLS* tumor lysis syndrome, *Y* yes.

Table 2 summarizes blood cell and cytokine levels pre- and blood cell and cytokine levels, toxicities and outcomes following infusion of CD33-CAR-T cells for all 3 treated patients. No dose-limiting toxicities (DLTs) were observed (Table 2) at dose level 1 during the 28-day DLT assessment period. Two patients experienced cytokine release syndrome (CRS) and 1 patient developed immune effector cell-associated neurotoxicity syndrome (ICANS). Adverse events possibly related to CD33-CAR-T cells included a grade 3 tumor lysis syndrome-acute kidney injury, grade 2 mucositis, grade 1 tachycardia for 1 patient; and a second patient experienced grade 2 intermittent orthostatic hypotension, grade 2 increased bilirubin and grade 3 increased ALT and AST. One patient experienced no toxicity. Adverse Events unrelated to the treatment were grade 3 respiratory distress syndrome in one patient and grade 3 febrile neutropenia and grade 4 sepsis and septic shock in another patient. All three patients who received CD33-CAR-T cells have died, due to disease progression.

All 3 patients had leukopenia on the day of CD33-CAR-T cell infusion; 2 had circulating blasts. Two methods were used to detect circulating CAR-T cells: flow cytometry with fluorescently labeled cetuximab and digital droplet polymerase chain reaction (ddPCR) for HER1t. CD33-CAR-T cells were detected by both methods. Patient 03 and 11 had CRS and had associated increase in blood IL-6 level. Despite the activity seen, none of the patients met criteria for response to treatment. There was in vivo evidence of CD33-CAR-T cells at this lowest dose level, which was associated with increase in inflammatory cytokine levels (IL6, IFNγ, TNFα) concurrent with ICANS and CRS, and for only those who manifested these symptoms (Table 2). No pediatric patients were enrolled due to closure of the trial upon clearing the first dose level with no DLT.

Due to challenges posed by the aggressive nature of relapsed/refractory AML and need for expeditious autologous product generation, logistic and enrollment challenges, the trial was closed to patient entry after patient 11 was enrolled and treated. The sponsors have transitioned to a platform that facilitates more rapid production and in vivo expansion with a product referred to as PRGN-3006 (NCT03927261) [9, 10].

We could detect CD33-CAR-T cells in blood samples from patients following infusion with associated symptoms and increased cytokine levels. Despite this biologic activity, we did not see anti-leukemia responses, although patients received only one dose of CD33-CAR-T cells, and this was the lowest dose level. A single case report of a patient with relapsed AML treated with CD33-CAR-T cells suggested therapeutic activity with associated symptoms and increase in inflammatory cytokine levels (NCT01864902) [11]. No further follow up has been provided for this trial. Our trial illustrated challenges associated with CAR-T therapy for patients with relapsed/refractory AML which relate to manufacturing using viral vectors, rapid disease progression and risk for infection, and coordination of administration of this therapeutic modality.

Most patients with relapsed/refractory AML have lymphopenia and many have increased number of circulating blood blasts, which complicate and increase difficulty of producing autologous CAR-T cells. In this clinical trial, there were 10 patients enrolled and the cell product meeting all prespecified release criteria was successfully made for 4 of these patients. Two enrolled patients were unable to undergo apheresis. Challenges included rapid disease progression and high blood blast count, lymphopenia and difficulty with acquiring adequate number of starting T cells in the apheresis step, difficulty of manufacturing using lentiviral vector, and disease progression or infection during lengthy production time, all of which contributed to inability to receiving product for some patients enrolled in this trial. Therefore, for patients with AML, there is need to develop a strategy which eliminates or markedly shortens manufacturing time to address the shortcomings of lentiviral transduction.

Relapsed/refractory AML is typically an aggressive disease with a rapid, unrelenting tempo of progression. Due to depletion of myeloid cells by the disease and treatments, patients are severely immunodeficient and at very high risk for infection. Considering the logistics and time of 2–4 weeks required for production and release with lentiviral and retroviral transduction, there is a significant time-at-risk for patients with respect to both progressive AML and infection. Events during this period represent a significant risk to receiving lymphodepletion and CAR-T cell product. “Bridging” chemotherapy has been used, but has associated toxicity and is usually myelosuppressive and immunosuppressive.

An ideal CAR-T cell target should to be easily accessible, expressed on the surface of all cancer cells and not be shared by viable tissues or normal hematopoietic cells, in order to reduce the eventual toxicity and side effects. AML antigen targets are usually also expressed on normal hematopoietic cells, which may result in undesired prolonged myelosuppression. Clinical trials are ongoing to test CAR-T cells targeting antigens other than CD33 on myeloid blasts including: CD123 (NCT03766126, NCT03114670, NCT03190278, NCT04230265, NCT03631576), IL1 receptor accessory protein (IL1RAP) (NCT04169022), CLL-1 (NCT04219163), CD38 (NCT04351022), and FLT-3 (NCT03904069). Despite expression on some normal myeloid progenitors, CD33 remains a reasonable and attractive therapeutic target. We observed in vivo CD33-CAR-T cells and activity and symptoms consistent with this observation. There are ongoing trials with CD33-CAR-T cell strategies (NCT03971799, NCT03927261).

In conclusion, autologous CD33-CAR-T cell production via lentiviral transduction was challenging, and was feasible only in patients with higher lymphocyte count and lower blood blasts. No DLTs were noted at the first dose level and in vivo CD33-CAR-T cells were detected. CRS and ICANS occurred, and were manageable for this lowest dose cohort. Higher T cell count at apheresis and rapid manufacturing strategies that reduce time from apheresis to treatment will be essential in CAR-T treatments for these challenging patients with relapsed/refractory AML.

## Supplementary information

Supplemental Material

Author Statement
