# Supplementary material for: Autologous CD33-CAR-T cells for treatment of relapsed/refractory acute myelogenous leukemia
Source: Leukemia. 2021 Apr 8;35(11):3282–6. doi: 10.1038/s41375-021-01232-2 (PMC8550958; doi:10.1038/s41375-021-01232-2)
Supplement: Supplementary file 1 — Supplemental Material [file 41375_2021_1232_MOESM1_ESM.docx]

**Supplemental Material**

**Autologous CD33-CAR-T Cells for Treatment of Relapsed/Refractory Acute Myelogenous Leukemia**

Francesco Paolo Tambaro, MD,PhD^1,2^

Harjeet Singh, PhD^1^

Emily Jones, RN^3^

Michael Rytting, MD^1^

Kris M. Mahadeo, MD^1^

Philip Thompson, MBBS^3^

Naval Daver, MD^3^

Courtney DiNardo, MD^3^

Tapan Kadia, MD^3^

Guillermo Garcia-Manero, MD^3^

Tim Chan, PhD^4^

Rutul R. Shah^4^

William G. Wierda, MD,PhD^3^

^1^Department of Pediatrics, The University of Texas MD Anderson Cancer Center, Houston, TX 77030

^2^Unità Operativa di Trapianto di Midollo Osseo e Servizio Trasfusionale; Azienda Ospedaliera di Rilievo Nazionale Santobono-Pausilipon, Napoli, Italy

^3^Department of Leukemia, The University of Texas MD Anderson Cancer Center, Houston, TX 77030

^4^Precigen, Inc.20358 Seneca Meadows Parkway, Germantown, MD 20876

Short title: CD33-CAR-T Cells for R/R AML

Corresponding Author:

William Wierda, MD, PhD

Department of Leukemia

The University of Texas MD Anderson Cancer Center

1515 Holcombe Boulevard, Unit 428

Houston, TX 77030

Phone: 713-745-0428; Fax: 713-794-1602

Email: [wwierda@mdanderson.org](mailto:wwierda@mdanderson.org)

**ABSTRACT**

Patients with relapsed/refractory acute myeloid leukemia (AML) have extremely poor prognosis, highlighting their need for new and novel treatments. Autologous T cells transduced to express chimeric antigen receptor (CAR) against CD19 have been a significant therapeutic advance for patients with relapsed/refractory non-Hodgkin lymphoma and acute lymphoblastic leukemia. Indeed, we aimed to target CD33 with autologous CAR-T cells for adult and pediatric patients with relapsed and refractory AML.

We initiated a phase I clinical trial of autologous T cells transduced with lentivirus vector to express CD33-directed second generation CAR and truncated epidermal growth factor receptor (kill switch), for adult and pediatric patients with relapsed/refractory AML.

Ten adults with relapsed/refractory AML were enrolled, 3 received a single dose of 0.3 x 10^6^ CD33-CAR-T cells/kg (lowest dose). All 3 had leukopenia at treatment; circulating transduced T cells were detected following infusion, and increases in inflammatory cytokines (IL6, IFNγ and TNFα) were observed. No dose limiting toxicities were observed; none of the patients met criteria for response.

We report feasibility challenges in this first dose cohort. Further clinical development must focus on shortening production time and improving CAR-T cell persistence may improve feasibility and efficacy of CD33-directed therapy for patients with relapsed/refractory AML.

**SUBJECTS AND METHODS**

We initiated a single-center, single-arm, Phase I clinical trial (NCT03126864) to investigate the feasibility and safety of autologous T cells, modified to express a CD33-targeted CAR with 4-1BB and CD3ζ endo-domains and co-expressed with truncated human epidermal growth factor receptor (HER1t)^1, 2^, in patients with relapsed/refractory AML. The goals of this Phase I clinical trial were to assess the feasibility and safety of adoptive transfer of the autologous CD33-CAR-T cells identify the recommended Phase II dose. T cells were *ex vivo* transduced to co-express the CD33-CAR and HER1t transgenes using a replication incompetent lentivirus (LV-CD33-CAR). Cetuximab, the mAb that targets epidermal growth factor receptor, can be administered as a safety measure to eliminate CAR-T cells in the event of significant toxicity. This CD33-CAR targeted the same CD33 binding epitope as gemtuzumab.

The primary objectives of this Phase I trial were to assess the feasibility and safety of adoptive cellular therapy employing *ex vivo* expanded autologous T cells, modified to express a CD33 targeted chimeric antigen receptor (CAR) and a truncated human epidermal growth factor receptor (HER1t) following lymphodepletion in patients with relapsed or refractory AML and to determine the recommended dose for Phase II evaluation. The window for assessment of dose limiting toxicities was the first 28 days after CD33-CAR-T infusion. A DLT was defined as a clinically significant adverse event or abnormal laboratory value assessed as unrelated to disease progression, intercurrent illness, or concomitant medications and occurring during the first course on study that met any of the following criteria: 1) CTCAE non-reversible grade 3, or any grade 4-5 allergic reactions related to the study cell infusion; 2) CTCAE non-reversible grade 3, or any grade 4-5 autoimmune reactions related to the study cell infusion; 3) CTCAE non-reversible grade 3, or any grade 4-5 organ toxicity (cardiac, dermatologic, gastrointestinal, hepatic, pulmonary, renal/genitourinary, or neurologic) not pre-existing or due to the underlying malignancy. Secondary objectives were to evaluate: 1) response (CR, CRp, CRn, CRi, PR or NR) to treatment; 2) immunogenicity of CD33-CAR-T cells through the detection of any anti-CD33-CAR-T antibody; 3) persistence of CD33-CAR-T cells 28 days post infusion; and 4) blood cell phenotypic and serum chemistries and electrolyte changes from baseline.

Adult (18 to 80 years) and pediatric (1 to <18 years) patients with active (blood or marrow blasts >5%) relapsed/refractory AML were planned to enroll in parallel cohorts. Patients must have bone marrow and peripheral blood studies available for confirmation of diagnosis of AML; CD33 positivity (>5% blasts CD33+) must be confirmed by either flow cytometry or immunohistochemistry; cytogenetics, flow cytometry, and molecular studies (such as FMS-like tyrosine kinase-3 [Flt-3] status) will be obtained as per standard practice. Patients who had undergone prior allogeneic stem cell transplantation (SCT) with no active GVHD and not on treatment or prophylaxis for graft-versus-host disease (GVHD) were eligible if they were at least 3 months post SCT. Additional inclusion criteria where: ECOG ≤2, pre-treatment creatinine clearance ≥ 60 mL/min, serum bilirubin ≤ 2 (except if Gilbert syndrome or liver involvement with leukemia, alanine aminotransferase (ALT) ≤ 2 normal levels, ejection fraction measured by echocardiogram (ECHO) or multi gated acquisition scan (MUGA) > 50%, oxygen saturation by pulse oximetry ≥94% in room air and no need of supplemental oxygen or mechanical ventilation, a negative pregnancy test, use of contraceptives for women of child-bearing potential and men. The following were exclusions: diagnosis of acute promyelocytic leukemia (APL); isolated extramedullary disease; known central nervous system (CNS) refractory to intrathecal chemotherapy or cranio-spinal radiation; ongoing or uncontrolled active infection; HIV, HBV or HCV infections; cardiac disease such as symptomatic congestive heart failure, unstable angina pectoris, cardiac arrhythmia; poorly controlled psychiatric illness/social situations limiting the compliance with the study requirements; presence of other malignancy within 2 years of study entry; pregnancy or breast feeding; history of allergic reaction attributable to cetuximab or similar compounds, or mouse and bovine proteins; patients receiving corticosteroids at >20 mg (age >17) or 0.5 mg/kg (age <18) daily prednisone or equivalent; patients with active autoimmune diseases requiring systemic immunosuppressive therapy were also excluded.

A “3+3” Phase I design with 3 dose levels of 0.3, 1, and 5 x 10^6^ CD33-CAR-T/kg for 2 parallel cohorts (adult and pediatric) was planned. The 1^st^ does cohort enrolled only adults. Starting with Dose Level 1, subjects were enrolled in a cohort size of 3. The dose escalation and recommended Phase II dose determination were conducted separately for the adult and pediatric patients based the standard 3+3 design. At each dose level, a cohort of three adult patients were to be treated initially. If at least 28 days had elapsed since the third adult received treatment at the first dose level and it was deemed as safe (i.e., with </=1 DLT), the pediatric cohort was to begin enrollment. If there were 2 or more DLTs in the Level 1 adult dose, the adults were to be de-escalated to the -1 Level and no pediatric cohort enrolled until after the -1 stage was completed with ≤ 1 DLT. If no dose level could be found in which there was 1 or less adult in 6 treated with DLT, then no pediatric subject was to be enrolled. Pediatric and adult cohorts were to be subsequently enroll and be assessed in parallel.

The clinical trial and informed consent were approved by the University of Texas MD Anderson Cancer Center IRB and the trial was conducted according to the Declaration of Helsinki. All patients provided signed informed consent, and were screened to confirm eligibility, then T cells were collected by apheresis for production. Patients received lymphodepletion with fludarabine 25 mg/m^2^ day -5 to -3 and cyclophosphamide 900 mg/m^2^ on day -3, followed by 2-days’ rest, then autologous CD33-CAR-T cells were administered. Bridging chemotherapy was allowed after leukapheresis and during the production phase of cell product at the discretion of treating physician.

Patients were monitored on the inpatient service for at least 10 days; their hospitalization was extended if they were experiencing continued symptoms of cytokine release syndrome (CRS) or Immune effector cell-associated neurotoxicity syndrome (ICANS). CRS was graded according to criteria and grading of Lee et al^3^. Clinical laboratories included daily CBC with differential, chemistries, and cytokine (IL6, IFNγ and TNFα) levels were performed in the clinical lab.

The autologous CD33-CAR-T cell product release criteria were according to Table S1 below.

Table S1

| **Testing Parameter** | **Acceptance Criteria** |
| --- | --- |
| Mycoplasma | Negative |
| Sterility^1^ | No growth on 7 Day sterility test |
| Endotoxin | ≤5 EU per kg |
| Cell Concentration | 1 - 3 x10^7^ cells/mL |
| Cell Viability | ≥ 70% |
| Purity (CD3^+^ T cells) | ≥ 80% |
| CD33-CAR expression | ≥ 10% |
| Residual CD3/CD28 beads | ≤ 34 beads/10^6^ cells |
| Replication Competent Lentivirus (RCL) | Negative |
| CAR gene copy number | 0.05-5 copies/cell |
| ^1^The cell product can be released based on a “no growth” result from 7 day sterility test (evaluated on or after Day 7). | |
| The sterility test will proceed for completion per USP <71> and the final result will be reported. | |

Presence of CAR and tumor in patient PBMCs after infusion was detected using CD33-Fc reagent (R&D Systems), cetuximab and anti-CD33 (Clone WM53, BD Biosciences)/anti-CD34 (Clone 8G12, BD Biosciences) antibodies respectively using flow cytometry. Data acquisition was on a BD LSRFortessa™ X-20 cell analyzer (BD Biosciences) using FACSDiva Software v 8.0.1 (BD Biosciences) and analyses of CD3-gated populations was undertaken using FlowJo Software v 9.7.6 (BD Biosciences).

Quantification of integrated CAR transgene in patient-derived serial peripheral blood samples after infusion was determined using droplet digital PCR (ddPCR). Genomic DNA from patient PBMCs along with SUP-T1 cell line expressing CD33-CAR-HER1t (as a positive control) and PBMCs without the transgene (as a negative control) was isolated using a commercial kit (Qiagen). PCR mixture containing genomic DNA, FAM labeled TaqMan primer/probe set (proprietary) was used to generate droplets, transferred to 96-well plates, amplified in a Bio-Rad C1000 Thermal Cycler (95^0^C 10min, 94^0^C 30sec, 58^0^C 30 sec, 98^0^C 10min) and evaluated using QX-100 Digital Droplet PCR system (Bio-Rad) according to the manufacturer’s instructions.

**REFERENCES**

1. Song D, Swartz MH, Tian L, Carvajal-Borda F, Plummer J, Shah RR*, et al.* Autologous T Cells Modified to Co-Express CD33-Specific Chimeric Antigen Receptor and a Kill Switch for Treatment of CD33+ Acute Myeloid Leukemia. *Blood* 2017; **130**(Supplement 1)**:** Abstract 1376.

2. Sallman D, Elmariah H, Sweet K, Talati C, Mishra A, Kelley L*, et al.* A Phase 1/1b Safety Study of Prgn-3006 Ultracar-T™ in Patients with Relapsed or Refractory CD33-Positive Acute Myeloid Leukemia and Higher Risk Myelodysplastic Syndrome. *Blood* 2020; **136**(Supplement 1)**:** (Abstract 2864).

3. Lee DW, Gardner R, Porter DL, Louis CU, Ahmed N, Jensen M*, et al.* Current concepts in the diagnosis and management of cytokine release syndrome. *Blood* 2014 Jul 10; **124**(2)**:** 188-195.
