# Supplementary material for: Autologous CD33-CAR-T cells for treatment of relapsed/refractory acute myelogenous leukemia
Source: Leukemia. 2021 Apr 8;35(11):3282–6. doi: 10.1038/s41375-021-01232-2 (PMC8550958; doi:10.1038/s41375-021-01232-2)
Supplement: Supplementary file 2 — Author Statement [file 41375_2021_1232_MOESM2_ESM.docx]

**Acknowledgements**

None

**Disclosure of Potential Conflicts of Interest**

FPT: No relevant conflicts of interest to report

HS: Research funding from Precigen/Intrexon

EJ: No relevant conflicts of interest to report

MR: No relevant conflicts of interest to report

KM: No relevant conflicts of interest to report

PT: No relevant conflicts of interest to report

ND: No relevant conflicts of interest to report

CD: No relevant conflicts of interest to report

TK: No relevant conflicts of interest to report

GGM: No relevant conflicts of interest to report

TC: Employee of Precigen/Intrexon

RRS: Employee of Precigen/Intrexon

WGW: No relevant conflicts of interest to report

**Author Contributions**

FPT: Analyzed and summarized data, wrote manuscript, reviewed and approved manuscript

HS: Correlative laboratory studies, data collection and summary, reviewed and approved manuscript

EJ: Research nurse, coordinated patient schedules, reviewed and approved manuscript

MR: Reviewed and assisted in writing protocol, reviewed and approved manuscript

KM: Collaborator, reviewed and approved manuscript

PT: Referred patients, collaborator, reviewed and approved manuscript

ND: Referred patients, collaborator, reviewed and approved manuscript

CD: Referred patients, collaborator, reviewed and approved manuscript

TK: Referred patients, collaborator, reviewed and approved manuscript

GGM: Referred patients, collaborator, reviewed and approved manuscript

TC: Performed pre-clinical work, reviewed and approved manuscript

RRS: Performed pre-clinical work, reviewed protocol, reviewed and approved manuscript

WGW: Designed and wrote protocol, enrolled and treated patients, oversaw data collection, supervised data analysis and summary and writing of manuscript, reviewed and approved manuscript
